# Modifiable Risk Factors, Health Profile and Well-Being of the Elderly Diagnosed with Cancer in Italy: Passi d’Argento Surveillance System 2016–2019 Results

**DOI:** 10.3390/cancers14246185

**Published:** 2022-12-14

**Authors:** Benedetta Contoli, Valentina Possenti, Valentina Minardi, Stefania Gori, Giordano Beretta, Maria Masocco

**Affiliations:** 1 National Centre for Disease Prevention and Health Promotion, Istituto Superiore di Sanità, 00161 Rome, Italy; 2 Unit of Medical Oncology, Sacro Cuore Don Calabria Hospital (IRCCS), 37024 Verona, Italy; 3 Unit of Oncology, Santo Spirito Hospital, 65122 Pescara, Italy

**Keywords:** elderly, cancer survivors, surveillance system, Italy, risk factors, epidemiology, prevention, health promotion, public health

## Abstract

**Simple Summary:**

Data 2016–2019 from the behavioral surveillance system PASSI d’Argento confirm the role of lifestyle-related factors in cancer survivorship and well-being among the elderly population residing in Italy. The data monitor different aspects of elderly life in terms of participation and social engagement, lifestyles and compliance with care and prevention activities. The analysis of how older individuals with cancer live is key to addressing effective prevention strategies, tailored to the specific needs of cancer survivors themselves. Facilitating access to social and health services for the most vulnerable groups would mean reducing health inequalities, accessing specific programs for the promotion of a healthy lifestyle, keeping chronic diseases under control to improve the well-being of the individual as well as of society. The study provides a useful basis for new models of multi-professional interventions to improve the health status among the elderly population living with cancer.

**Abstract:**

(1) Cases of cancer are expected to increase in the next years and the risk of cancer increases with age. Data 2016–2019 from the Italian population-based surveillance PASSI d’Argento (PdA) allow the description of the physical and psychosocial well-being of people aged ≥65 years diagnosed with cancer (Ca), and the comparison with elderly suffering from other chronic conditions (Ch) and healthy older individuals (H). (2) Data are collected by Local Health Units’ professionals using a standardized questionnaire during telephone interviews. (3) A total of 8051 out of the 56,352 interviewees reported a previous diagnosis of cancer: an annual average cancer prevalence of 12.8% (95% CI 12.4–13.3%) corresponding to 1.725 million elderly residing in Italy. In comparison to the H, Ca were more likely to refer bad health (aPR = 4.21; 95% CI: 3.70–4.79), suffer from depressive symptoms (aPR = 2.65; 95% CI: 2.35–2.99), disability (aPR = 2.50; 95% CI: 2.22–2.81) or sensory problems (aPR = 1.51; 95% CI: 1.40–1.63), be frail (aPR = 1.45; 95% CI: 1.30–1.61). Ca are often current smokers (aPR = 1.26; 95% CI: 1.11–1.45) and sedentary (aPR = 1.10; 95% CI: 1.03–1.18). (4) PdA provides valuable information to researchers and policy-makers by showing the difficulties for older people with cancer in contributing socially and accessing basic social and health services, which amplifies the risk of cognitive decline, isolation, and psychological deterioration.

## 1. Introduction

Over the past ten years, the age-adjusted incidence of cancer and mortality rates of the elderly with cancer have been declining in the majority of high-income countries. Nonetheless the complete prevalence (generally measured in absolute numbers and proportions) has been increasing constantly since early 2000, as a result of the ageing population and a higher survival rate, owing to early diagnosis and improved treatment options [1]. In Italy, cancer-related mortality is lower than the European average (−13% in men and −10% in women). In 2021, a decreasing trend of deaths and a better cancer survivorship were both observed and, more in general, between 2015 and 2021 mortality rates for all cancers reached a 10% and 8% decrease, respectively, among men and women. However, the total deaths increased by 0.6% (males) and 2.0% (females), achieving 100,200 and 81,100 deaths in the two sexes, which reflects the constant ageing population. In 2020, the number of people with a cancer diagnosis was 3.6 million which is 6% of the whole population living in Italy, with a 36% increase compared to the estimates projected in 2010. [2]. The elderly are particularly affected: already in 2010 in Italy almost four out of ten of those previously diagnosed with cancer were over 75 years old, and in this age group, the complete prevalence of cancer was 17% whereas it was less than 1% under 45 [1]. Complete cancer prevalence includes patients currently treated for cancer; those who have become cancer free, but are still at risk of recurrence or death; and, finally, patients with death rates similar to those of the general population who can be considered “cured patients”. Many of these individuals may suffer physical, cognitive, and/or psychosocial limitations and understanding the medical and psychosocial needs of cancer survivors is crucial to assessing and managing these issues actively [3]. Several studies have explored extensively the correlation between lifestyle behavioral factors and altered nutrient metabolism or other mechanisms, providing wide epidemiologic evidence on the suggested application of lifestyle intervention for cancer risk reduction, prevention, and control strategies [4,5,6,7]. Conversely, less attention is however dedicated to the prevalence and effects of behavioral risk factors on health-related well-being among cancer survivors [3]. Indeed, lifestyle factors are increasingly being implicated in cancer prognosis: obesity, inactivity, poor dietary quality, alcohol consumption, and continued smoking after a cancer diagnosis have all been linked to increased risk of cancer recurrence and mortality in individuals with common cancers [8]. Lifestyle-related behaviors (e.g., weight management, physical activity, diet, and so on) play a vital role in the health of cancer survivors. As the population of cancer survivors increases, health professionals across diverse settings will become more involved in cancer survivorship care. Based on the scientific evidence that has shown how certain lifestyle behaviors can enhance the lives of cancer survivors and, in some cases, reduce cancer-related mortality, numerous cancer organizations have developed guidelines for assessing lifestyle-related factors among cancer survivors along with evidence-based counseling strategies to help motivate patients toward healthier behaviors [9].

This study aims to describe behavioral risk factors and physical, psychological, and social well-being profiles of people aged 65 years and older living in Italy, who have been diagnosed with cancer at least once, to identify priorities for strategies to maintain the highest attainable standard of health among current or former cancer patients. For this purpose, we used data from PASSI d’Argento (PdA) [10,11,12], the ongoing nationwide population-based surveillance system of residents in Italy for 65 years of age and over, which continuously collects data on health status and health behaviors associated with the most common chronic diseases, participation in society and employment, independent living, safety, and living environment. PdA surveillance system, since 2016, monitors several conditions to identify the need for further action for health promotion within the public health national service. The availability of PdA data offers the opportunity to analyze a large age-and-gender representative sample of older people living in Italy, specifically taking a picture of the health status of those who have been diagnosed with cancer at least once in their lifetime, comparing it, as well as possible, with profiles of people of the same age, with or without chronic conditions.

## 2. Materials and Methods

### 2.1. Data Source (Design, Setting) and Population under Study

The PdA population-based surveillance system monitors continuously a wide range of health-related behaviors as well as the physical and psychological health and social conditions of people aged 65 and over in Italy. PdA is based on cross-sectional surveys by ongoing data collection at the Local Health Unit (LHU) level. The target population consists of all people aged 65 and over residing in the LHU area. The eligible population comprehends residents who may be contacted telephonically and are capable of being interviewed whereas those hospitalized, residents in nursing homes and prisoners at the time of the interview are not eligible. Those who do not speak Italian are also excluded except in the autonomous province of Bolzano, where interviewees have the option of being interviewed in German.

In each LHU, a random sample is periodically drawn from the LHU’s list of residents, classified according to sex and age (65–74; 75–84, 85+) proportionally to the respective sex-age group in the general population. Specially trained personnel from the LHU carry out telephone interviews using a standardized questionnaire of about 80 closed-ended questions. The LHUs’ data are merged and analyzed to obtain national and regional estimates. The national coordination center at the Italian National Institute of Health (namely, Istituto Superiore di Sanità) provides the Ministry of Health with annual reports of aggregated data to inform decisions makers on priorities for national health policies regarding prevention and health promotion strategies. Detailed information on the PdA sampling methodology can be found elsewhere [12].

Except Lombardy and Valle d’Aosta, all Regional Authorities participated in PdA in 2016–2019. A total of 56,352 interviews of people over 65 years of age have been collected, with a response rate of over 86% and a refusal rate of 10%.

#### 2.1.1. Case Definition

In PdA, the over 65 respondents are asked if, over the lifetime, they had ever been diagnosed with cancer or other chronic diseases such as diabetes, kidney failure, chronic bronchitis, emphysema, asthma, respiratory insufficiency, stroke myocardial infarction, coronary and other heart diseases, tumors, chronic liver disease or cirrhosis.

Hereinafter, “*Older people with cancer*” (Ca) are identified as interviewees who reported that a physician has diagnosed cancer, including leukemia and lymphomas, once in their lifetime.

“*Older individuals with chronic diseases other than cancer*” (Ch) are defined as interviewees who reported that a physician has diagnosed at least one of the following diseases: kidney failure, chronic bronchitis, emphysema, respiratory failure, bronchial asthma, stroke or cerebral ischemia, diabetes, myocardial infarction, cardiac ischemia or coronary disease, other heart diseases such as heart failure or valvulopathy, chronic liver disease, cirrhosis.

For the purposes of this analysis, interviewees who did not report any of the above-mentioned diseases are considered as “*Healthy older persons*” (H).

#### 2.1.2. Variables Definition

The older people engaging in the PdA interview answered questions on the following aspects describing their physical, psychological, and social well-being:

(1) Health–health perception, unhealthy days (the overall number of days out of the previous 30 days during which the respondent felt that either their own physical or mental health was not good or had activity limitations due to poor physical or mental health), mental well-being (depressive symptoms as identified by the Patient Health Questionnaire 2, PHQ-2) [13,14,15], problems with eyesight, chewing and hearing, falls, frailty, and disabilities (assessed by the Katz Index of Independence in Activities of Daily Living, ADL, and the Lawton Instrumental Activities of Daily Living, IADL). In particular, disability indicates the lack of self-sufficiency in one of the six ADLs—bathing, dressing, toileting, moving, continence and feeding—while frailty defines the difficulties that the elderly without disabilities have in two out of eight ADLs—making phone calls, shopping, preparing food, housekeeping, laundry, transportation, self-medicating, managing finances, [16,17];
(2) Prevention and health promotion: modifiable risk factors (tobacco smoking, high alcohol consumption (more than one alcoholic unit per day), physical inactivity according to the 2010 World Health Organization recommendation [18] (using the Physical Activity Scale for Elderly-PASE [19,20] questions), insufficient fruit and vegetable consumption (less than five portions per day), obesity (BMI ≥ 30), complying with professional advice on correct lifestyles (giving up smoking, reducing alcohol consumption, engaging in regular physical activity), seasonal influenza vaccination uptake;
(3) Social Participation and Security: The importance of being an asset to people living with them or to others doing voluntary/charity activities, engaging in paid work, being socially connected, attending training courses; having access to local health, social and commercial services, living in a safe neighborhood.
  The 2016–2019 PdA participants reported their sociodemographic characteristics: gender, age, educational level (none or primary school, high school, university), economic difficulties (many, some, or none at all), nationality (Italian or other), and geographic residence area according to the National Institute of Statistics criteria (North, Centre, South and major islands).

### 2.2. Statistical Analysis

Based on the PdA data gathered in the timeframe 2016–2019 we calculated: (i) the prevalence of the three subgroups indicated above (Ca, Ch, and H) overall and by socio-demographic criteria; (ii) the prevalence of indicators about physical, psychological, and social well-being among Ca, Ch, and H to obtain their health and social profile.

Finally, to compare the profiles of Ca’s health and lifestyles to the other two groups, we performed Poisson regression analyses on specific health outcomes (poor health perceived, depressive symptoms, frailty, disability, sensory problems) and on modifiable risk factors (smoking—current and former, excessive alcohol consumption, physical inactivity, low fruit and vegetable consumption and obesity).

Adjusted prevalence ratios (aPRs) for age, gender, educational level, economic difficulties, and macro area of residence, are presented with corresponding 95% confidence intervals (95% CI) and *p*-value. The aPRs on health outcomes are also adjusted for comorbidity (presence of two or more chronic diseases).

Prevalence data are computed with confidence intervals at 95% (95% CI).

Complex survey design analyses were conducted, using the Taylor series method to estimate variance. The statistical package StataCorp LP 16.1 software supplied the data analysis.

## 3. Results

In 2016–2019, 56,352 interviews of persons over 65 years of age were collected by the PdA. We excluded 325 questionnaires with missing data on chronic diseases. Overall, 56,027 respondents were included in the analyses: 8051 reported a previous cancer diagnosis (4779 with comorbidity and 3272 without), 25,901 reported one or more chronic diseases ≠ cancer, and 22,075 reported no chronic diseases.

### 3.1. Prevalence of Cancer, Chronic Disease and Healthy People

Table 1 reports the prevalence estimated by PdA of cancer, presence, and absence of chronic disease.

The 8051 out of the 56,027 PdA interviewees reported a previous diagnosis of cancer amounts to annual average cancer prevalence of 12.8% (95% CI 12.4–13.3%), corresponding to 1.7 million people aged ≥65 years residing in Italy. The Ca percentage does not differ significantly between men (13.2%) and women (12.6%), it ranges from 11.4% among people aged 65–74 to 14.5% in the 75–84 year-old age group and subsequently decreases to 13.4% from 85 years old and above. It is significantly higher among people living in the Northern and Central Regions (16.0% and 14.2%, respectively) than in Southern Italy (9.3%). The prevalence of cancer is higher among people who report economic hardship in making ends meet with their finances available (14.6% compared to 12.5–12.9% among people with some or no economic difficulties).

Among the interviewees, 48% (25,901 interviewees) reported a chronic disease other than cancer, and the remaining 39% (i.e., 22,075 interviewees) could be classified as healthy (the equivalent of 6662 million and 5259 million people aged ≥65 years residing in Italy respectively). As expected, the prevalence of chronic diseases increases with age-up to 59.3% among the over 85-year-olds, whereas prevalence decreases with age in healthy subjects. Only 27.3% of the over 85-year-olds have not been diagnosed with cancer or chronic diseases among those studied in PdA. Men suffer chronic conditions more than women do (50.7% vs. 45.8%). The prevalence of chronic diseases is higher among people residing in southern regions (56.0% compared to the 46.3% in the centre and 39.8% in the north) and among people with serious economic difficulties (62.3% compared to 52.7% or 40.3% among people with relative or no economic difficulties and a low level of education (53.7% and 42.0% respectively).

### 3.2. Health Status, Prevention and Social Participation in the Elderly Diagnosed with Cancer

Table 2 shows the prevalence of health status, preventing profile and social participation indicators among the three identified groups of people aged ≥65 years (with cancer, with chronic disease other than cancer, and healthy).

Among Ca, 23.6% reported poor overall state of health (compared to 16.8% among Ch and 5% of H). They stated that in more than 14 of the past 30 days both their physical and psychological health was poor (respectively, 23.9% and 19.6%), and their activity was limited in 16.2% of cases. A percentage of 20.2% reported depressive symptoms according to the PHQ-2. These figures are significantly higher than those observed among Ch (with the exception of the days with limited activity) and well above those among H. Ca report sensory problems (i.e., problems with seeing 11.9%, hearing 15.6%, and chewing 14.5%) which are not resolvable by using specific devices. These problems are also common among Ch (respectively, 13.9%, 18.7%, 17.9%), but less frequent in H (5.4%, 8.6%, 6.7%). Ca (9.7%) and also Ch (10.2%) reported falls within the previous month significantly more frequently than the H (6.2%). Ch resulted to be the group at major risk of frailty (23.4%) in comparison with Ca (19.6%) and H (11.7%), whereas the risk of disability is similar in Ca (20.5%) and Ch (20.9%) compared to 6.5% in H.

Health-related behavior among Ca seem similar to that of the rest of the population (with or without chronic diseases). Unhealthy behavior is even more frequent among Ca: of which 11.0% smoke currently (vs. 9.7 among Ch and 9.6% among H) and 31.9% are former smokers; 18.1% refer high-risk alcohol consumption (17.2% among Ch and 20.2% among H); physical inactivity prevails in 39.8% (vs. 44.8% in Ch and 34.2% in H); obesity is 14.8% (vs. 17.1% among Ch and 10.7% among H) and 12.3% follow the “five a day” recommendation (i.e., an intake of at least five portions of fruit and vegetables per day).

Healthcare professionals’ advice is more focused on smoking (76.2% of Ca who smoke are advised to quit compared to 75.9% among Ch and 58.6% in H), but are less inclined to suggest on the one hand at-risk drinkers to reduce their alcohol intake (12.5% among Ca and 13.4% respectively Ch and 7.8% in H) and on the other hand inactive people to increase their physical activity (30.3% Ca, 28.8% Ch, 25.5% H). Individuals aged ≥65 years rarely comply with seasonal influenza vaccination uptake: overall, only 56.4% of the elderly resident population received the annual flu vaccination over the last 12 months, this rises to 59.5% in Ca and to 63.9% among Ch, but falls to 46.3% in H.

People aged 65 years and over are considered an asset in 28.3% of cases among Ca, 24.0% in Ch and 34.7% if H, to people living with them (Ca 17.6%, Ch 16.7%, 22,4% H) and to others (respectively 13.8%, 11.6%, 17.8%). To a lesser extent they do voluntary work (Ca 5%, Ch 3.8%, H 7.1%) or paid work (respectively 5.2%, 5.1%, 8.0%). Ca have also the lowest rates of social connectivity (20.0% vs. 23.2% among Ch and 28.7% among H) and attending training courses is particularly rare with a prevalence of 4.4% in Ca, 3.7% in Ch, and 6.1% in H.

Ca and especially by Ch refer difficulties in accessing essential social and health services. As far as healthcare services are concerned, barriers to reach general practitioners (GPs) are reported respectively by 26.7% of Ca, 31.2% of Ch, and 13.1% of H; to access pharmacies by 26.0% Ca, 30.8% Ch, 12.7% H; these difficulties increase in accessing LHU services (33.6% in Ca 39.0% in Ch 18.5% in H). Even harder to reach are municipal services (Ca 31.9%, Ch 37.3%, H 17.0%) together with shopping centers and supermarkets (34.3% in Ca, 40.6% in Ch, 19.7% in H). Ca perceived a lower level of safety in their neighborhood (18.1%) than Ch (16.0%) and H (12.0%) do.

Table 3 and Table 4 show the aPRs for health profile and modifiable risk factors among people aged ≥65 years with cancer compared to people with chronic disease other than cancer and healthy individuals.

As shown in Table 3, the multivariate analysis confirms that, controlling by comorbidity, gender, age, geographic area, educational level, and economic conditions the overall psychophysical health profile is somewhat compromised in Ca, in particular when compared to H: Ca are three times more likely to refer bad health (aPR = 3.09; 95% CI: 2.59–3.69), almost two more likely to suffer from depressive symptoms (aPR = 2.12; 95% CI: 1.80–2.51) or disability (aPR = 1.83; 95% CI: 1.49–2.26), and also have sensory problems (aPR = 1.26; 95% CI: 1.12–1.43) or to be frail (aPR = 1.45; 95% CI: 1.22–1.71). Health perception and depression symptoms among Ca are worse than those observed in Ch.

In Table 4 the multivariate analysis shows that also unhealthy lifestyles (except for alcohol drinking and fruit and vegetables consumption) are more frequent in Ca compared to H and very similar to Ch, who would also benefit from lifestyle changes. Compared to H, Ca are more likely to be former smokers (aPR = 1.30; 95% CI: 1.22–1.39) or even current smokers (aPR = 1.26; 95% CI: 1.11–1.45), featured by a sedentary lifestyle (aPR = 1.10; 95% CI: 1.03–1.18).

## 4. Discussion

In PdA ’people with cancer’ among the over 65s, as people who have been diagnosed with cancer once in their lifetime, are identified by asking respondents whether a doctor has ever diagnosed them with cancer and which cannot be verified by objective diagnostic data. Thus, the PdA results, based on the self-reported medical diagnosis of cancer, can be considered an approximate estimate of the prevalence of cancer among the elderly living in Italy. Nevertheless, they are consistent with those made by the Italian Cancer Registries. According to this study, 2.6 million people were estimated to be living in Italy in 2010 after a cancer diagnosis, with complete cancer prevalence estimates increasing with age from less than 1% below age 45 to 12.1% at age 65–74, 16.6% at 75–84 and 16.7% above 85. The forecast for 2020 indicated an increase in prevalent cancer cases to 3.6 million people, of whom 1.5 million were over 75 [1]. 

According to PdA estimates, the complete cancer prevalence on annual average from 2016–2019 corresponds to 11.4% at 65–74 years, 14.5% between 75–84 years of age and 13.5% at over 85, i.e., the equivalent of 1.8 million people among the population aged ≥65 years resident in Italy in 2020, of which one million are over 75 years of age (applying these estimates to the residing population as provided by the National Institute of Statistics—ISTAT) on 1 January 2020 [21]. 

Estimates from PdA are consistent, but slightly lower than those in the Guzzinati et al. study, particularly in people over 85 years of age. This could be due to the fact that the PdA eligible population includes residents able to be interviewed and living at home, and therefore excluding people in poor health unable to answer the questionnaire and people who are hospitalized or living in nursing homes at the time of the interview [12]. Despite this limitation, analysing the PdA database (i.e., a nationally representative sample of the general population aged 65+ living in Italy), it was possible to describe the profile of the physical, psychological and social well-being of elderly who have probably survived some form of cancer in their lifetime and are currently living at home.

Perceived health, psychological well-being, sensory problems as well as independence in daily living (frailty and disability) are significantly impaired among the over-65s diagnosed with cancer, compared to healthy people. When compared to people with chronic diseases, those with cancer were more likely to have depressive symptoms and bad perceived health. 

Unhealthy lifestyles such as smoking, high alcohol consumption, being physically inactive, or consuming little fruit and vegetables, are all significant risk factors for cancer recurrence or even worsening the oncologic pathology itself [22,23,24,25,26].

Nevertheless multivariate analysis shows that unhealthy behaviors as currently smoking, physical inactivity and obesity are more frequent in Ca in comparison to H and overall similar to Ch.

The intake of fruit and vegetables remains insufficient and comparable in all three groups. Only excessive alcohol consumption is less frequent among Ca and Ch than among H, regardless of the fact that the consumption of alcohol, even in moderate quantities, in old age is not without risk, in particular when chronic diseases are also present.

Healthcare workers advice, a proven effective mean of contrasting unhealthy behaviours and promoting healthy habits, is not widely used, even among patients diagnosed with cancer. Particular low attention is given to physical inactivity and alcohol consumption. As also reported in the literature, it appears there are few barriers to raising the topics of diet, smoking and alcohol—yet body weight and exercise are mentioned much less frequently [27]. On the one hand, typical patient-related barriers include being “too busy” and a “lack of willpower”—although barriers can vary between cancer types and patient groups [28]. On the other hand, reluctance to discuss these important lifestyle factors appears to arise from a desire to minimise patient distress—but limitations in health providers’ knowledge and their perceptions of the quality and strength of evidence about lifestyle factors also appear to be important. The emphasis in overcoming these barriers has been on developing high-reach, sustainable interventions which can reach very broad and diverse populations—this is particularly needed if studies are to include sufficient subgroups to ensure generalisability of findings. Incorporation of the identified barriers when developing health professionals training programmes and lifestyle interventions could increase the probability of successful behavioural change, and thus improve outcomes for cancer survivors [29]. Haussmann et al. undertook a qualitative study examining factors which might hinder health professionals in recommending physical activity in their patients with cancer. Health professionals were concerned with the physical overexertion and psychological stress which might arise from a recommendation for physical activity. Other factors included the physical and social environments of the patient with cancer and the availability of exercise programmes. It is important for health professionals to consider physical activity recommendations, taking into account patient circumstances—and to not be “put off” by factors which there may have little relevance to their patient’s circumstances [30]. Del Valle and Colleagues examined dietary behaviours in women with breast cancer—they found that a relatively simple intervention was effective in bringing about sustainable improvements in diet, such as greater consumption of fruit and vegetables and lower fat intake. These findings add to the growing body of evidence that simple, cost-effective lifestyle interventions can bring about dietary changes in patients with cancer—changes which are sustainable well beyond the life of the intervention [31]. 

Another shortcoming within the preventive measures targeting the elderly is the low influenza immunization uptake far from reaching the national public health goals. Flu vaccination which should cover all people over 65 and all those with cancer, is also far from being at its best [32]. This low coverage of elderly people living in Italy corresponds with the same figure in Europe (44% in 2017) [33].

Participating and being socially engaged as well as contributing to economic, cultural and civic activities are fundamental to preserve healthy and active ageing [34]. In Europe, it is also clear how much voluntary work contributes to promoting healthy ageing and intergenerational solidarity, a significant role being played by associations and non-governmental organizations [35,36]. However, compared to the rest of the population, being part of a society has significant limitations in people diagnosed with cancer or other chronic diseases.

To avoid the risk of isolation, older people need to be able to easily reach certain services, such as the general practitioner and/or the local LHU, pharmacies and supermarkets and shops for basic needs, and they need to be able to feel safe in their daily routine in the neighborhood. Instead, besides fewer occasions of engaging in social life, people diagnosed with cancer experience difficulties in gaining access to essential services and more frequently than others perceive their neighborhood as insecure.

Facilitating access to social and health services for the most vulnerable would mean reducing the inequalities of health, having access to specific programs for the promotion of a healthy lifestyle, keeping chronic diseases under control to improve the well-being of the individual and society. Participating less frequently in society, difficulty in accessing social and health or essential services and an insecure living environment increase the risk of isolation in people diagnosed with cancer, with the well-known implications that this can have on psychological well-being and cognitive decline [37,38,39].

### Limitations

A main strength of the study is that it relies on a large and representative sample of the older population living in Italy. The PdA design aims in fact to monitor over time the physical, psychological, and social well-being profile of the elderly aged 65 and over, by collecting data on a wide range of behavior and health-related conditions during old age. Such surveillance systems allows the dynamic description of these aspects because of monitoring their changes over time, and also identifying the most vulnerable groups and detecting social inequalities regarding health and prevention. However, certain limitations cannot be ignored.

*Self-reporting*. First, as per any interview-based health survey, PdA data (except for the main demographic characteristics: gender, age, residence) as well as information on cancer diagnosis are self-reported. Although socially undesirable behaviors are likely to be underestimated, self-reported data on cancer and/or chronic diseases seem to be adequately consistent and sensitive enough to have an important role within public health resources. Being ever diagnosed or confirmed any chronic disease including cancer refers to a relevant and rare behavior and is not prone to misclassification or recall bias.

*Lack of information*. From PdA no information is available on age at diagnosis or period after having been diagnosed, site or type of cancer; moreover, no information is available on the treatment received. For this reason, Ca “Older people with cancer” include patients with treatment of cancer ongoing, patients who had a diagnosis of cancer within one year, five years and longer.

Nevertheless, this limitation does not compromise the main objective of the study that was to describe Ca behavioral risk factors and psychological and social well-being profiles in order to identify Ca health needs and priorities for action aimed at maintaining the highest attainable standard of health and quality of life.

*Unit nonresponse*. Because nonrespondents often differ from interviewees for characteristics under consideration, high nonresponse rates can lead to inaccurate measures of the indicators. There is no general consensus about which value is acceptable because the alteration of the estimates depends on many factors, such as survey design, study population, reasons for nonresponse, explored variables. However, the PdA sample is extracted from the LHU lists of residents and this procedure translates to a much wider coverage than similar surveys (e.g., the American BRFSS) so that, as indicated in the Methods’ section, the final response rate is >86%, and then, pretty well assessed. Lastly, the results cannot fully reflect the profile of all elderly people diagnosed with cancer since those living in nursing homes or hospitals, who presumably have a worse health profile, are ineligible to PdA [12].

## 5. Conclusions

In Italy, the constantly increasing aging of the population is significantly straining the capacity and performance of the public health and social systems and services. Furthermore, the COVID-19 pandemic further exposed the vulnerability of and lack of capacity within the Italian public health systems to care for older persons. Since its start, the findings from PdA have been providing useful information to health professionals, researchers, and policy-makers by identifying both high-priority issues and relevant healthcare gaps for older people. More in detail, the availability of data even on how people with cancer live is crucial to better address effective prevention strategies, tailored onto the specific needs of cancer survivors.

Health perception, physical and psychological well-being, and achieving a sufficient level of independence to carry out daily activities are all undoubtedly compromised in people reporting a previous diagnosis of cancer, even more so than in people with other chronic diseases.

Among people with cancer, as well as among people with chronic diseases, the prevalence of health risk behaviors is far from optimal. Unhealthy lifestyles are major risk factors for cancer recurrences or even aggravate the oncologic pathology itself.

Additionally, the advice provided by health professionals is little used to change behavioral risk factors in people with cancer, when it would be so important to reduce the several cancer-related risks: recurrence, primary tumors in other sites or just for the management of the disease itself. Even the influenza vaccination coverage that should concern all over 65-year-olds and cancer patients is instead far from the threshold.

The PdA data show the relevant number of people with cancer who experience difficulties in contributing socially and gaining access to essential services; all of which amplify the risk of cognitive decline, isolation, and the negative repercussions on psychological well-being. This biopsychosocial health profile of people with cancer in Italy emphasizes to what extent healthy behavior and appropriate health and social policies could improve the quality of life and health needs of this specific group of the population, and therefore guarantee the right to the highest attainable standard of health among current or former cancer patients. In this framework, our study may allow a basis for reasoning about new models of lifestyle intervention to improve care and well-being of older patients with cancer and their health outcomes across care-continuum, by identifying challenges specific to interprofessional care practice and to all involved in planning cancer services.

## Figures and Tables

**Table 1:** Distribution of sample (n = 56,027) and prevalence of three groups of people aged ≥65 years (with cancer, with chronic disease other than cancer, and healthy) by socio-demographical characteristics. Passi d’Argento 2016–2019.

			Prevalence of People with:					
	Distribution of Sample (n = 56,027)		Cancer (n = 8051)		Chronic Disease ≠ Cancer (n = 25,901)		Healthy (n = 22,075)	
	n	%	%	(95% CI)	%	(95% CI)	%	(95% CI)
**Total**	**56,027**	**-**	**12.84**	**(12.39–13.30)**	**47.93**	**(47.22–48.64)**	**39.24**	**(38.55–39.93)**
**Age Troup**								
65–74	26,913	49.6	11.43	(10.86–12.03)	41.64	(40.67–42.63)	46.92	(45.93–47.92)
75–84	20,185	35.8	14.54	(13.70–15.41)	52.02	(50.82–53.21)	33.45	(32.35–34.56)
≥85	8929	14.6	13.44	(12.31–14.66)	59.26	(57.38–61.12)	27.30	(25.63–29.04)
**Gender**								
Men	24,464	43.3	13.17	(12.47–13.91)	50.71	(49.65–51.77)	36.12	(35.11–37.13)
Women	31,563	56.7	12.58	(12.00–13.18)	45.80	(44.86–46.75)	41.62	(40.69–42.56)
**Geographic Area of Residence**								
North	28,154	37.1	16.01	(15.19–16.87)	39.82	(38.66–40.99)	44.17	(42.99–45.36)
Centre	9706	21.2	14.20	(13.23–15.23)	46.29	(44.82–47.76)	39.51	(38.10–40.94)
South	18,167	41.7	9.32	(8.73–9.93)	55.97	(54.88–57.06)	34.71	(33.68–35.76)
**Economic Difficulties**								
Many	4947	11.2	14.65	(13.20–16.22)	62.32	(60.29–63.31)	23.03	(21.42–24.72)
Some	18,272	38.5	12.46	(11.70–13.27)	52.74	(51.55–53.93)	34.79	(33.68–35.92)
None	30,958	50.3	12.85	(12.24–13.47)	40.32	(46.84–48.29)	46.84	(45.83–47.85)
**Education Level**								
Low (primary school or none)	25,554	48.8	12.60	(11.93–13.31)	53.7	(52.63–54.75)	33.7	(32.70–34.73)
High (Middle/High school or University)	29,584	51.2	13.26	(12.65–13.89)	42.0	(40.98–42.92)	44.8	(43.83–45.76)

**Table 2:** Prevalence of health status, preventing profile and social participation indicators among three groups of people aged ≥65 years (with cancer, with chronic disease other than cancer, and healthy). Passi d’Argento 2016–2019 (n = 56,027).

		People with Cancer (n = 8051)		People with Chronic Disease ≠ Cancer (n = 25,901)		Healthy People (n = 22,075)	
		%	(95% CI)	%	(95% CI)	%	(95% CI)
Health profile		23.65	(21.91–25.48)	16.83	(16.03–17.65)	5.00	(4.51–5.54)
	+14 unhealthy days (physical health)	23.86	(22.14–25.68)	19.62	(18.69–20.58)	8.95	(8.23–9.71)
	+14 unhealthy days (psychological health)	19.56	(17.96–21.26)	16.44	(15.56–17.36)	8.49	(7.66–9.39)
	+14 unhealthy days (daily activity limitations)	16.16	(14.73–17.7)	14.70	(13.86–15.58)	4.69	(4.21–5.22)
	Depressive symptoms (PHQ-2)	20.18	(18.56–21.9)	17.59	(16.68–18.55)	7.03	(6.42–7.7)
	Sight impairment	11.92	(10.81–13.13)	13.89	(13.19–14.62)	5.43	(4.94–5.97)
	Hearing loss	15.59	(14.38–16.88)	18.65	(17.88–19.44)	8.63	(8.05–9.25)
	Chewing problems	14.50	(13.18–15.93)	17.92	(17.1–18.76)	6.69	(6.19–7.23)
	Fall (within the previous 30 days)	9.68	(8.63–10.84)	10.22	(9.53–10.97)	6.22	(5.71–6.77)
	Frailty (at least 2 IADL)	19.64	(18.19–21.17)	23.40	(22.52–24.31)	11.73	(11.02–12.48)
	Disability (at least 1 ADL	20.49	(18.89–22.19)	20.88	(20.04–21.75)	6.46	(5.89–7.08)
Modifiable Risk Factors	Currently smoking	11.03	(9.82–12.36)	9.67	(9.05–10.32)	9.56	(8.95–10.21)
	Formerly smoking	31.93	(30.20–33.71)	28.44	(27.55–29.35)	23.62	(22.71–24.56)
	At-risk alcohol consumption	18.11	(16.64–19.68)	17.17	(16.41–17.95)	20.21	(19.35–21.09)
	Physical inactivity	39.80	(37.67–41.98)	44.84	(43.59–46.09)	34.17	(33.02–35.35)
	Five a day	12.37	(11.25–13.57)	9.84	(9.27–10.44)	12.50	(11.79–13.25)
	Obesity	14.78	(13.52–16.12)	17.13	(16.32–17.98)	10.74	(10.02–11.5)
Health Promotion: Professionals’ Advice	To quit smoking	76.21	(70.82–80.88)	75.93	(72.86–78.75)	58.61	(54.96–62.17)
	To reduce alcohol consumption (to at-risk drinkers)	12.48	(9.05–16.97)	13.43	(11.9–15.12)	7.76	(6.39–9.39)
	To engage in physical activity	30.31	(28.63–32.05)	28.85	(27.89–29.83)	25.53	(24.50–26.59)
Protective behaviors	Influenza immunization *	59.48	(57.64–61.3)	63.91	(62.9–64.91)	46.28	(45.14–47.42)
Social participation	Being an asset	28.29	(26.67–29.97)	24.04	(23.16–24.93)	34.66	(33.57–35.76)
	To people living with them	17.56	(16.16–19.05)	16.68	(15.9–17.49)	22.36	(21.44–23.32)
	To people not living with them	13.84	(12.60–15.17)	11.64	(10.99–12.33)	17.85	(17.02–18.71)
	Voluntary activities	5.01	(4.33–5.79)	3.80	(3.44–4.19)	7.11	(6.41–7.87)
	Paid work	5.20	(4.53–5.97)	5.12	(4.63–5.66)	7.96	(7.43–8.53)
	Social connectedness	20.00	(19.18–20.85)	23.18	(22.57–23.8)	28.65	(27.56–29.76)
	Participation in training courses	4.41	(3.78–5.15)	3.71	(3.32–4.16)	6.10	(5.62–6.61)
	Neighbourhood security	18.15	(16.43–20)	16.00	(15.16–16.87)	12.05	(11.3–12.85)
Difficulty in accessing services	General Practitioner	26.71	(25.08–28.4)	31.18	(30.21–32.16)	13.12	(12.35–13.92)
	Local Health Unit services	33.60	(31.81–35.43)	39.01	(37.98–40.04)	18.54	(17.64–19.48)
	Pharmacy	25.95	(24.33–27.63)	30.76	(29.79–31.75)	12.67	(11.9–13.47)
	Municipality services	31.86	(30.11–33.65)	37.25	(36.24–38.28)	16.96	(16.11–17.85)
	Commercial services	34.33	(32.49–36.21)	40.62	(39.6–41.65)	19.68	(18.76–20.62)

* PASSI d’Argento interviewees report compliance with seasonal flu vaccination in the previous year.

**Table 3:** Health profile among people aged ≥65 years with cancer compared to people with chronic disease other than cancer and healthy individuals. Passi d’Argento 2016–2019 (n = 56,027). Adjusted prevalence ratios–aPRs (by Poisson regression models) for comorbidity, age, gender, educational level, economic difficulties and regional macroarea with corresponding 95% confidence intervals (95% CI) and *p*-value.

		People with Cancer (n = 8051) vs. Healthy People (n = 22,075)			People with Cancer (n = 8051) vs. People with Chronic Disease ≠ Cancer (n = 25,901)		
	Outcomes	aPR	(95% CI)	*p* Value	aPR	(95% CI)	*p* Value
Health profiles	Perceived Health (bad)	3.09	(2.59–3.69)	*p* < 0.01	1.35	(1.23–1.48)	*p* < 0.01
	Depressive symptoms	2.12	(1.80–2.51)	*p* < 0.01	1.16	(1.05–1.29)	*p* < 0.01
	Frailty	1.45	(1.22–1.71)	*p* < 0.01	0.92	(0.84–1.01)	n.s. *
	Disability	1.83	(1.49–2.26)	*p* < 0.01	1.07	(0.98–1.17)	n.s. *
	Sensory factor	1.26	(1.12–1.43)	*p* < 0.01	0.95	(0.89–1.01)	n.s. *

* n.s. not significant.

**Table 4:** Modifiable risk factors among people aged ≥65 years with cancer compared to people with chronic disease other than cancer and healthy individuals. Passi d’Argento 2016–2019 (n = 56,027). Adjusted prevalence ratios–aPRs (by Poisson regression models) for age, gender, educational level, economic difficulties and regional macroarea with corresponding 95% confidence intervals (95% CI) and *p*-value.

		People with Cancer (n = 8051) vs. Healthy People (n = 22,075)			People with Cancer (n = 8051) vs. People with Chronic Disease ≠ Cancer (n = 25,901)		
	Outcomes	aPR	(95% CI)	*p* Value	aPR	(95% CI)	*p* Value
Modifiable Risk Factors	Currently smoking	1.26	(1.11–1.45)	*p* < 0.01	1.13	(0.99–1.23)	n.s.*
	Formerly smoking	1.30	(1.22–1.39)	*p* < 0.01	1.07	(1.01–1.14)	*p* < 0.05
	At-risk alcohol consumption	0.89	(0.82–0.97)	*p* < 0.01	1.00	(0.91–1.09)	n.s. *
	Physical inactivity	1.10	(1.03–1.18)	*p* < 0.01	0.95	(0.89–1.01)	n.s. *
	Five a day	1.04	(0.93–1.17)	n.s. *	1.10	(0.98–1.23)	n.s. *
	Obesity	1.02	(1.00–1.03)	*p* < 0.05	0.96	(0.95–0.98)	*p* < 0.01

* n.s. not significant.

## Data Availability

The data presented in this study are available on request from the corresponding author. The data are not publicly available due to restrictions, e.g., privacy or ethical.

## References

[1] Guzzinati S., Virdone S., De Angelis R., Panato C., Buzzoni C., Capocaccia R., Francisci S., Gigli A., Zorzi M., Tagliabue G. (2018). Characteristics of people living in Italy after a cancer diagnosis in 2010 and projections to 2020. BMC Cancer.

[2] Associazione Italiana di Oncologia Medica (2021). I Numeri del Cancro in Italia 2021.

[3] Choi J.W., Hua T.N.M. (2021). Impact of Lifestyle Behaviors on Cancer Risk and Prevention. J. Lifestyle Med..

[4] Cao Z., Xu C., Yang H., Li S., Wang Y. (2021). The Role of Healthy Lifestyle in Cancer Incidence and Temporal Transitions to Cardiometabolic Disease. JACC Cardio Oncol..

[5] Zhang Y.B., Pan X.F., Chen J., Cao A., Zhang Y.G., Xia L., Wang J., Li H., Liu G., Pan A. (2020). Combined lifestyle factors, incident cancer, and cancer mortality: A systematic review and meta-analysis of prospective cohort studies. Br. J. Cancer.

[6] Arem H., Loftfield E. (2018). Cancer Epidemiology: A Survey of Modifiable Risk Factors for Prevention and Survivorship. Am. J. Lifestyle Med..

[7] Spector D. (2018). Optimizing Cancer Survivors’ Health: The Role of Lifestyle Behaviors. JNP.

[8] Ligibel J. (2012). Lifestyle factors in cancer survivorship. J. Clin. Oncol..

[9] DeSantis C.E., Lin C.C., Mariotto A.B., Siegel R.L., Stein K.D., Kramer J.L., Alteri R., Robbins A.S., Jemal A. (2014). Cancer treatment and survivorship statistics. CA Cancer J. Clin..

[10] La Sorveglianza PASSI d’Argento EpiCentro. https://www.epicentro.iss.it/passi-argento.

[11] Decree of the President of the Council of Ministers of 3 March 2017. Identification of surveillance systems and registers of mortality, cancer and other diseases. (17A03142) (OJ General Series No.109 of 12-05-2017).

[12] Contoli B., Carrieri P., Masocco M., Penna L., Perra A., PDA Study Group (2016). PASSI d’Argento (Silver Steps): The main features of the new nationwide surveillance system for the ageing Italian population, Italy 2013–2014. Ann. Ist. Super Sanita.

[13] Kroenke K., Spitzer R.L., Williams J.B. (2003). The Patient Health Questionnaire-2: Validity of a two-item depression screener. Med. Care.

[14] Löwe B., Kroenke K., Gräfe K. (2005). Detecting and monitoring depression with a two-item questionnaire (PHQ-2). J. Psychosom. Res..

[15] Thombs B.D., Benedetti A., A Kloda L., Levis B., Nicolau I., Cuijpers P., Gilbody S., A Ioannidis J.P., McMillan D., Patten S.B. (2014). The diagnostic accuracy of the Patient Health Questionnaire-2 (PHQ-2), Patient Health Questionnaire-8 (PHQ-8), and Patient Health Questionnaire-9 (PHQ-9) for detecting major depression: Protocol for a systematic review and individual patient data meta-analyses. Syst. Rev..

[16] Wallace M., Shelkey M. (2007). Hartford Institute for Geriatric Nursing. Katz index of independence in activities of daily living (ADL). Urol. Nurs..

[17] Lawton M.P., Brody E.M. (1969). Assessment of older people. Self-maintaining and instrumental activities of daily living. Gerontologist.

[18] World Health Organization Physical Activity. Key Facts. 26 November 2020. https://www.who.int/news-room/fact-sheets/detail/physical-activity.

[19] Washburn R.A., Smith K.W., Jette A.M., Janney C.A. (1993). The physical activity scale for the elderly (PASE): Development and evaluation. J. Clin. Epidemiol..

[20] Washburn R.A., McAuley E., Katula J., Mihalko S.L., Boileau R.A. (1999). The physical activity scale for the elderly (PASE): Evidence for validity. J. Clin. Epidemiol..

[21] ISTAT Demografia in Cifre. https://demo.istat.it/.

[22] Kroenke C.H., Fung T.T., Hu F.B., Holmes M.D. (2005). Dietary patterns and survival after breast cancer diagnosis. J. Clin. Oncol..

[23] Chan D.S.M., Vieira A.R., Aune D., Bandera E.V., Greenwood D.C., McTiernan A., Navarro Rosenblatt D., Thune I., Vieira R., Norat T. (2014). Body mass index and survival in women with breast cancer-systematic literature review and meta-analysis of 82 follow-up studies. Ann. Oncol..

[24] Playdon M.C., Bracken M.B., Sanft T.B., Ligibel J.A., Harrigan M., Irwin M.L. (2015). Weight Gain After Breast Cancer Diagnosis and All-Cause Mortality: Systematic Review and Meta-Analysis. J. Natl. Cancer Inst..

[25] Meyerhardt J.A., Giovannucci E.L., Ogino S., Kirkner G.J., Chan A.T., Willett W., Fuchs C.S. (2009). Physical activity and male colorectal cancer survival. Arch. Intern. Med..

[26] Ballard-Barbash R., Friedenreich C.M., Courneya K.S., Siddiqi S.M., Mc Tiernan A., Alfano C.M. (2012). Physical activity, biomarkers, and disease outcomes in cancer survivors: A systematic review. J. Natl. Cancer Inst..

[27] Miles A., Simon A., Wardle J. (2010). Answering patient questions about the role lifestyle factors play in cancer onset and recurrence: What do health care professionals say?. J. Health Psychol..

[28] Ottenbacher A.J., Day R.S., Taylor W.C., Sharma S.V., Sloane R., Snyder D.C., Kraus W.E., Demark-Wahnefried W. (2011). Exercise among breast and prostate cancer survivors—What are their barriers?. J. Cancer Surviv..

[29] Koutoukidis D., Lopes S., Fisher A., Williams K., Croker H., Beeken R.J. (2018). Lifestyle advice to cancer survivors: A qualitative study on the perspectives of health professionals. BMJ Open.

[30] Haussmann A., Gabrian M., Ungar N., Jooß S., Wiskemann J., Sieverding M., Steindorf K. (2018). What hinders healthcare professionals in promoting physical activity towards cancer patients? The influencing role of healthcare professionals’ concerns, perceived patient characteristics and perceived structural factors. Eur. J. Cancer Care.

[31] Del Valle M.O., Martín-Payo R., Cuesta-Briand B., Lana A. (2018). Impact of two nurse-led interventions targeting diet among breast cancer survivors: Results from a randomized controlled trial. Eur. J. Cancer Care.

[32] Piano Nazionale Prevenzione Vaccinale 2017–2019. Gazzetta Ufficiale della Repubblica Italiana del 18/02/2017. Serie Generale—n.41. https://www.trovanorme.salute.gov.it/norme/renderPdf.spring?seriegu=SG&datagu=18/02/2017&redaz=17A01195&artp=1&art=1&subart=1&subart1=10&vers=1&prog=001.

[33] Eurobarometer (2019). 44% of Elderly People Vaccinated against Influenza. https://ec.europa.eu/eurostat/web/products-eurostat-news/-/ddn-20191209-2.

[34] World Health Organization Brasilia Declaration on Ageing. 1–3 July 1996. https://apps.who.int/iris/bitstream/handle/10665/330624/WH-1997-Jul-Aug-p20-21-eng.pdf.

[35] Eurobarometer (2011). Volontariato e Solidarietà Intergenerazionale.

[36] Eurobarometer (2012). Active Ageing.

[37] Kuiper J.S., Zuidersma M., Oude Voshaar R.C., Zuidema S.U., van den Heuvel E.R., Stolk R.P., Smidt N. (2015). Social relationships and risk of dementia: A systematic review and meta-analysis of longitudinal cohort studies. Ageing Res. Rev..

[38] Douglas H., Georgiou A., Westbrook J. (2017). Social participation as an indicator of successful aging: An overview of concepts and their associations with health. Aust. Health Rev..

[39] Loyola W.S., Camillo C.A., Torres C.V., Probst V.S. (2017). Effects of an exercise model based on functional circuits in an older population with different levels of social participation. Geriatr. Gerontol. Int..

